# The clinical relevance of WDFY4 in autoimmune diseases in diverse ancestral populations

**DOI:** 10.1093/rheumatology/keae183

**Published:** 2024-03-20

**Authors:** Xia Lyu, Janine A Lamb, Hector Chinoy

**Affiliations:** Department of Rheumatology, Renji Hospital, Shanghai Jiaotong University School of Medicine, Shanghai, China; Division of Musculoskeletal and Dermatological Sciences, Faculty of Biology, Medicine and Health, University of Manchester, Manchester, UK; Epidemiology and Public Health Group, School of Health Sciences, University of Manchester, Manchester, UK; Epidemiology and Public Health Group, School of Health Sciences, University of Manchester, Manchester, UK; Division of Musculoskeletal and Dermatological Sciences, Faculty of Biology, Medicine and Health, University of Manchester, Manchester, UK; Department of Rheumatology, Salford Royal Hospital, Northern Care Alliance NHS Foundation Trust, Manchester Academic Health Science Centre, Salford, UK

**Keywords:** WDFY4, GWAS, cross-presentation, anti-MDA5 antibody positive dermatomyositis, RP-ILD, biomarker

## Abstract

WD repeat- and FYVE domain-containing protein 4 (WDFY4), coded by a gene on 10q11.23, is a member of the BEACH (Beige and Chediak-Higashi) domain-containing family. Genome-wide association studies identified *WDFY4* variants as a risk factor for SLE in Asian and European populations. *WDFY4* variants are also associated with RA and primary biliary cholangitis, in different ancestry populations. The WDFY4 protein plays an essential role in the cross-presentation of classic dendritic cells, reactive oxygen species-induced apoptosis of CD8+ T cells, and non-canonical autophagic activity in B cells. A novel variant rs7919656 was identified in Japanese clinically amyopathic dermatomyositis patients, with a highly expressed truncated isoform augmenting the melanoma differentiation-associated gene 5 (MDA5) signalling pathway. The same variant was later found to be significantly associated with RP-ILD in Chinese MDA5+DM patients. Here, we briefly review the association of *WDFY4* with autoimmune diseases and its known function in the immune response.

Rheumatology key messages

- WDFY4 was identified by GWAS as a risk factor for SLE, RA, and PBC worldwide.
- WDFY4 functions in cross-presentation, T-cell apoptosis, and B-cell autophagy.
- WDFY4 polymorphism has the potential to predict RP-ILD in myositis.

## Introduction

The pathogenesis of autoimmune diseases is complex. Genetically susceptible individuals who are exposed to environmental triggers and develop immune intolerance is a common model. Genome-wide association studies (GWAS) have identified thousands of loci in the human genome associated with autoimmune disease, many of which are in the HLA region or in genes known to be involved in immune pathways [1]. At the same time, novel genes have also been identified by GWAS, such as WD repeat- and FYVE domain-containing protein 4 (*WDFY4*). Recently, *WDFY4* variant rs7919656 was reported to be associated with rapidly progressive interstitial lung disease (RP-ILD) in anti-melanoma differentiation-associated gene 5 autoantibody-positive dermatomyositis (MDA5+DM) [2], could be a promising tool to predict poor prognosis in newly diagnosed MDA5+DM patients and thus influence management strategy. In this review, we will discuss the *WDFY4* gene structure, functional roles, and association with autoimmune diseases, including myositis.

## WDFY4 gene structure and related gene families

**Figure 1.**
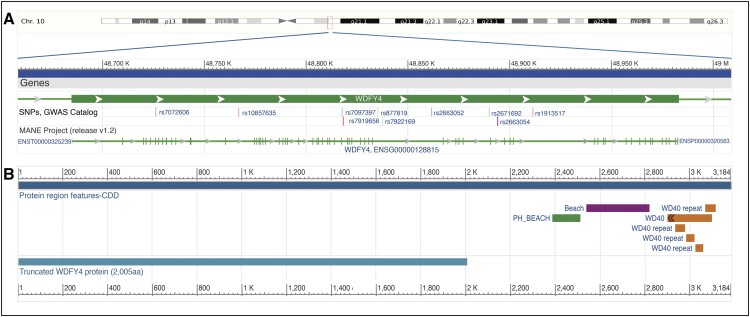
*WDFY4* gene location on chromosome 10 and the conserved motifs in WDFY4 protein. A. Gene location, structure, and autoimmune disease related SNPs. *WDFY4* (Ensembl [3]: ENSG00000128815) is located on 10: 48 684 873–48 982 956 (GRCh38). 10 SNPs are reported to be associated with autoimmune diseases in GWAS Catalog database [6]. B. Protein regions and truncated protein structure. The longest canonical transcript (ENST00000325239.20) selected by MANE Project [5] encodes the protein (ENSP00000320563.5) with 3184 amino acids, including a PH-domain, BEACH domain and 5 WD40 repeats. rs7919656 variant could introduce splicing, resulting in a 6018 bp transcript and finally a truncated isoform with 2005 amino acids [7]. The truncated-WDFY4 isoform has no PH, BEACH or WD domains

WD repeat- and FYVE domain-containing protein 4 (*WDFY4*, Ensembl [3]:ENSG00000128815), previously known as *C10orf64*, is a protein-coding gene located on 10q11.23, or more precisely 10:48684873–48982956 (GRCh38) [3]. Multiple transcripts have been detected in humans according to Ensembl [3] and RefSeq [4] data (Supplementary Table S1, available at *Rheumatology* online), and the canonical transcript (ENST00000325239.20) selected by MANE Project [5] contains 62 exons and encodes a large protein (ENSP00000320563.5) consisting of 3184 amino acids (Fig. 1).

WDFY4 is a member of the BEACH (Beige and Chediak-Higashi) domain-containing family, named after a rare and lethal autosomal recessive disorder, Chediak-Higashi syndrome (CHS), and its animal model *Beige_j_* mouse [8]. CHS patients are characterized by recurrent infections resulting from severe immunodeficiency, bleeding tendency, albinism, and progressive neurologic dysfunction [9]. The mutated gene responsible for CHS is *CHS1/LYST*, which encodes the protein lysosomal trafficking regulator (LYST).

Originally identified in the LYST protein, the BEACH domain is a conserved sequence enriched in the amino acids tryptophan, isoleucine, aspartic acid, and leucine. The BEACH domain is followed by a WD-40 domain, containing highly conserved repeating units usually ending with tryptophan-aspartic acid (WD) [10]. BEACH domain along with WD-40 repeats defines the BEACH domain-containing proteins (BDCPs) family. There are nine BDCPs in humans, including LYST, WDFY4, WD, and FYVE zinc finger domain-containing protein 3 (WDFY3), neurobeachin, neurobeachin-like 1, neurobeachin-like 2, lipopolysaccharide responsive beige-like anchor protein, neutral sphingomyelinase activation-associated factor and WD repeat domain 81. Like most other BDCPs, WDFY4 contains a pleckstrin homology (PH) domain preceding the consensus BEACH and WD40 domains (Fig. 1B). Interestingly, although it is called WDFY4, it does not contain a complete FYVE domain (named after four proteins), which is different from WDFY3 [8].

## Genome-wide association studies identify susceptibility variants within *WDFY4* in systemic lupus erythematosus and other autoimmune diseases

In 2009, a GWAS performed on SLE patients from a Chinese Han population reported nine new susceptibility loci [11], including one single nucleotide polymorphism (SNP) rs1913517 on chr 10q (Table 1). The study subjects were recruited from multiple hospitals in central and southern China, including an initial cohort with 1047 SLE cases and 1205 healthy controls. Two replication studies were conducted to validate the SNPs identified in the initial cohort, and in total 4188 SLE cases and 8255 controls were included in the combined study. The frequency of rs1913517 risk allele A was significantly higher in SLE cases compared with healthy controls (0.33 *vs* 0.29, odds ratio (OR) = 1.24, 95% Confidence Interval (CI) 1.17–1.32, *p*= 7.22 × 10^−12^, in the combined study). rs1913517 is located within an intron of two protein-coding genes, *WDFY4* (coded by the forward strand) and *LRRC18* (leucine-rich repeat containing 18, coded by the reverse strand).

**Table 1.** *WDFY4* variants associated with autoimmune diseases (based on GWAS Catalog [6], until Jan 2024)^a^

Variant	Location	A1	A1 frequency	P-value	OR	95%CI	Traits	Case number	Control number	Ancestry	Reference	Study accession
rs7072606	10:48725929 (missense variant)	T	0.81	2.22 × 10^−12^	0.88	0.85-0.92	SLE	13377	194993	East Asian	[12]	GCST011956
rs10857635	10:48766611 (intrinsic)	A	0.67	6.87 × 10^−10^	1.16	1.10-1.22	SLE	6748	11516	European	[13]	GCST005752
			0.78					2970	2452	African American		
			0.77					1872	2016	Hispanic		
rs7097397	10:48817351 (missense variant)	G	0.37^a^	8.15 × 10^−12^	1.3	1.21-1.40	SLE	2944	3655	East Asian	[14]	GCST000592
			0.42					314	519	South East Asian		
			0.35	8.69 × 10^−12^	1.2	NA	SLE	5201	9066	European	[15]	GCST003156
			0.37	2.10 × 10^−9^	1.33	1.21-1.46	SLE	1174	4246	East Asian	[16]	GCST003599
			0.38	8.6 × 10^−11^	1.22	NA	SLE	4036	6959	European	[17]	GCST003622
		A	0.65	2.23 × 10^−40^	0.81	0.78-0.84	SLE	13377	194993	East Asian	[12]	GCST011956
			0.66	1.4 × 10^−12^	0.92	0.90-0.94	RA	14361	43923	European	[18]	GCST90013534
			0.19					8267	244741	East Asian		
			0.4	1.43 × 10^−12^	0.93	0.91-0.95	RA	22350	74823	European	[19]	GCST90132222
			0.69					11025	162608	East Asian		
			0.1					999	1108	African		
			0.35					986	1258	South Asian		
			NA					511	352	Arab		
			0.69^b^	1.34 × 10^−10^	0.89	0.86-0.92	RA	11025	162608	East Asian	[19]	GCST90132224
			0.4	1.01 × 10^−10^	0.93	0.90-0.95	RA Seropositive	17221	74823	European	[19]	GCST90132225
			0.69					8340	162608	East Asian		
			0.1					841	1108	African		
			0.35					689	1258	South Asian		
			NA					357	352	Arab		
			NA	2.42 × 10^−10^	0.14 unit decrease^c^	0.10-0.19	PBC	8021	16489	European	[20]	GCST90061440
rs7919656	10:48817975 (intrinsic)	A	0.36	1.5 × 10^−8^	3.87	2.23-6.55	CADM	33	6270	East Asian	[7]	GCST005338
rs877819	10:48834906 (intrinsic)	A	0.21	8.1 × 10^−9^	1.32/1.46^d^	NA	SLE	1656	3394	East Asian	[21]	GCST001795
			NA	2.73 × 10^−9^	NA	NA	SLE	1639	2410	East Asian	[22]	GCST90020042
rs7922169	10:48837411 (intrinsic)	T	NA	5.47 × 10^−8^	0.11 unit increase^c^	0.07-0.15	PBC	8021	16489	European	[20]	GCST90061442
								2495	4283	East Asian		
rs2663052	10:48861350 (intrinsic)	C	0.45	5.25 × 10^−9^	1.16	1.10–1.22	SLE	5201	9066	European	[15]	GCST003155
rs2671692	10:48889774 (intrinsic)	A	0.65	2.8 × 10^−9^	1.07	1.05-1.10	RA	18136	49724	European	[23]	GCST002318
			0.31					8766	22439	East Asian		
			0.68	2.66 × 10^−8^	NA	NA	RA	868	1194	European	[24]	GCST007843
rs2663054	10:48893932 (intrinsic)	G	0.58	1.49 × 10^−8^	1.2	1.14-1.28	SLE	6748	11516	European	[13]	GCST007400
rs1913517	10:48911009 (intrinsic)	A	0.33	7.22 × 10^−12^	1.24	1.17-1.32	SLE	4199	8255	East Asian	[11]	GCST000507

A total of 10 SNPs surpassed the genome-wide significance threshold of *p* ≤ 5 × 10^−8^. Variant locations, mapped to Genome Assembly GRCh38, are annotated with their corresponding gene functional regions, specifically to the MANE select transcript, indicated in brackets, based on Ensembl(3) data. A1, the effect allele tested in the study. Ancestral nomenclature follows the framework applied by GWAS Catalog [25].

a Frequency is calculated based on data from different cohorts.

b Frequency from 1000 genome project [26].

c The value here is β value.

d Frequency is calculated based on two East Asian cohorts; ORs are the data of two cohorts respectively.

OR: odds ratio; CI: confidence interval; PBC: primary biliary cholangitis; CADM: clinically amyopathic dermatomyositis; NA: data not available.

Published at a similar time, a GWAS study in different Asian populations also identified novel variants in *WDFY4* associated with SLE [14, 27]. Conducted by the Asia Lupus Genetics Consortium and collaborating institutions, the study first genotyped 314 SLE patients and 1484 healthy controls in a Hong Kong Chinese population. Twenty SNPs in the *WDFY4* gene showed significant association with SLE, including the SNP rs1913517 mentioned above. Further replication studies of selected SNPs were conducted in four independent cohorts from Hong Kong, Shanghai, Anhui (China), and Bangkok (Thailand), with a total of 3300 Asian patients and 4200 ancestrally and geographically matched controls. rs7097397 and rs877819 were significantly associated with SLE, although only rs7097397 remained significant after conditional logistic regression (Table 1). The rs7097397 risk allele G (OR = 1.30, 95% CI 1.21–1.40, *p* = 8.15 × 10^−12^, in joint analysis), leads to a missense change in exon 31 of *WDFY4*, substituting the arginine residue for a glutamine (R1816Q).

Following these two studies, the association of SNPs located within *WDFY4* with SLE was validated not only in East and South East Asian cohorts but also in European populations (Table 1). Both SNPs rs1913517 and rs7097397 were confirmed by stepwise regression analysis in a Korean GWAS(16] including 1174 SLE cases and 4246 healthy population controls (rs7097397: OR = 1.33, 95% CI 1.21–1.46, *p* = 2.10 × 10^−9^; rs1913517: OR = 1.24, 95% CI 1.12–1.37, *p* = 2.54 × 10^−5^). This study also tried to validate the association of SNP rs877819, but no significant association was replicated. Haplotype analysis indicated that rs7097397 accounts for the association observed at rs1913517, and the risk alleles of rs7097397 and rs1913517 are in strong linkage disequilibrium (LD) with the major allele of rs877819, which may explain the above findings [16]. In 2013, Wang *et al.* [28] reported the genotyping results of SNP rs1913517 in three independent European cohorts recruited from Sweden, Finland, and the United States. A total of 2676 SLE patients and 10 162 controls were included, and rs1913517 was significantly associated with SLE in the meta-analysis (OR = 1.16, 95% CI 1.08–1.23, *p* = 7.8 × 10^−6^). Interestingly, although the direction of effect was consistent, the minor allele G of rs1913517 in European cohorts corresponded to the major allele in East and South East Asians.

Another study including 7219 cases and 15 991 controls of European ancestry reported a novel SNP rs2663052 in *WDFY4*, in strong LD (*r*^2^ = 0.7) with rs7097397, associated with SLE susceptibility in a meta-analysis (OR = 1.16, 95% CI 1.10–1.22, *p* = 5.25 × 10^−9^) [15] (Table 1). Notably, an expression quantitative trait loci (eQTL) mapping study based on the same GWAS data suggested two possible mechanisms involving *WDFY4* [29]. Missense variant rs7097397 (R1816Q) was the most likely functional variant, according to the functional annotation, while another putative splicing mechanism was also discovered. Cis-eQTL analysis showed that rs2663052 is correlated with upregulation of the exon 34A–34B junction of *WDFY4*, which is unique to a short isoform (552 amino acids) lacking the two WD40 functional domains essential for enzymatic activity [29]. Another missense variant within the *WDFY4* locus (rs7072606, S214P) was identified in East Asian ancestry SLE populations, with an effect independent of known rs7097397 (pairwise LD *r*^2^ = 0.021) [12]. GWAS conducted in European, African American, and Hispanic ancestry SLE populations identified another two SNPs, *WDFY4* rs2663054 and rs10857635 [13] that were significantly associated with the disease (Table 1).

*WDFY4* variants are also associated with other autoimmune diseases, including RA and primary biliary cholangitis (PBC), in different ancestry cohorts. Missense variant rs7097397 of *WDFY4* (R1816Q) was confirmed to be associated with the risk of RA in multi-ancestry populations [18, 19]. Consistent with the GWAS findings in SLE, allele A is the major allele in East Asian populations but the minor allele in European populations and has protective effects on RA development. *WDFY4* rs2671692 variant was also reported to be associated with trans-ethnic RA populations [23, 24] (Table 1). A genome-wide meta-analysis confirmed that *WDFY4* rs7097397 was associated with PBC in European ancestry populations, and rs7922169 in a combined analysis of European and East Asian populations [20] (Table 1).

Overall, although the allele frequency of multiple variants in the *WDFY4* gene in different ancestry populations varies and shows different linkage disequilibrium patterns (Supplementary Fig. S2, available at *Rheumatology* online), 10 variants associated at genome-wide significance (*p* ≤ 5 × 10^−8^) have been reported consistently associated with autoimmune diseases according to the GWAS Catalog database [6] (Table 1; Fig. 1A). Functional annotation and expression studies have identified candidate causal variants, supporting a role for WDFY4 in autoimmune disease risk.

## Function of WDFY4: a role in cross-presentation and lymphocyte activation

Although WDFY4 came to prominence more than ten years ago, its function remained unknown for a long time and is still not fully elucidated.

In 2018, the function of WDFY4 was first reported in classical dendritic cell (cDC) mediated cross-presentation [30]. The immune system typically employs two mechanisms of antigen presentation. One involves binding endogenous antigens with MHC-I molecules and presenting them to cytotoxic CD8+ T cells. The other involves the uptake of exogenous antigens into cells, binding with MHC-II molecules, and presenting them to CD4+ helper T cells. Cross-presentation is a distinctive mode of antigen presentation, by which exogenous antigens captured by cDCs are degraded into peptides and then loaded onto MHC-I molecules through the vacuolar or cytosolic pathway [31]. The peptide-MHC class I complex is then transferred to the surface of cDCs to recruit and activate naive CD8+ T cells, resulting in the production of cytotoxic T cells to kill the pathogen-infected cells without antigen-presenting function [32].

**Figure 2.**
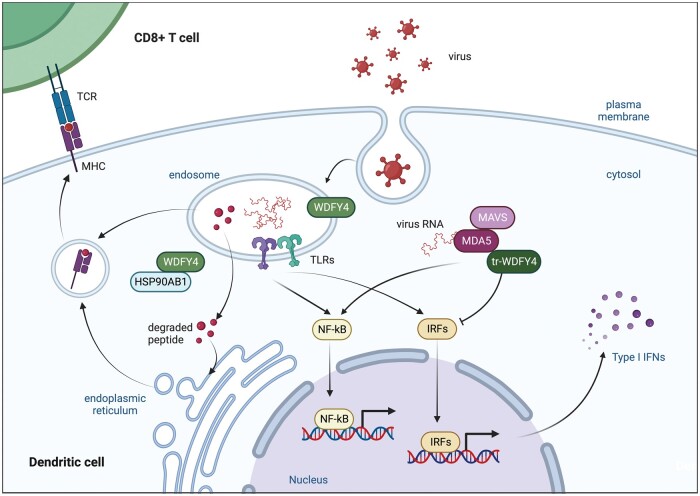
The possible functions of WDFY4. In virus infected dendritic cells, WDFY4 plays a role in trafficking between the cell surface and endosomes, and possibly interacts with HSP90AB1 to release antigen from the endosome to the cytosol, which is a part of cross-presentation to CD8+ T cells [30]. In vitro experiments also showed that both canonical WDFY4 protein and a truncated isoform caused by *WDFY4* rs7919656 variant can enhance the NF-κB signalling of pattern recognition receptors, including TLR3/TLR4/TLR9 and MDA5 [7]. The truncated WDFY4 isoform is reported to interact with MDA5 in the cytosol and change downstream signalling. TLR, Toll-like receptor. NF-κB, Nuclear factor-kappa B. IRF, interferon regulatory factor. IFN, interferon. MDA5, melanoma differentiation-associated gene 5. MAVS, mitochondrial antiviral signalling protein. HSP90AB1, heat shock protein 90 alpha family class B member 1. (image created with BioRender.com)

Theisen *et al.* [30] designed a functional clustered regularly interspaced short palindromic repeats (CRISPR) screen for candidate regulators of cross-presentation, and *WDFY4* was identified to be responsible for impaired cross-presentation in cDC1, a subset of cDCs that mainly control CD8+ T cell-related anti-viral and anti-tumour type 1 immune response. Although cDC1s developed normally and had the capacity to present endogenous antigens on MHC class I complexes in *Wdfy4^-/-^* mice, gene ontology analysis and *in vivo* experiments indicated that WDFY4 functions in assembling protein complexes and subcellular vesicular trafficking, possibly interact with heat shock protein 90 alpha family class B member 1, a member of the Heat Shock Protein 90 chaperone family involved in endosome-to-cytosol translocation of antigen during cross-presentation (Fig. 2). Further functional examination showed *Wdfy4^-/-^* cDC1s were only impaired in cross-presentation to CD8+ T cells, but not in priming of CD4+ T cells. Also, MHC class II antigen processing was unchanged in *Wdfy4*^–/–^ mice for both cell-associated and soluble antigens, and MHC class II antigen processing by B cells was also normal in *Wdfy4*^–/–^ mice, which indicates that the function of WDFY4 is restricted to MHC-I. Theisen *et al.* [30] also demonstrated that cDC2s, the other subset of cDCs mediating the cross-presentation of pathogen antigens, were not influenced by the loss of Wdfy4, indicating that the antigen presented by WDFY4 involved cross-presentation is restricted to an exogenous tumor or viral antigens. Although the cellular mechanism for WDFY4 activity remains unknown, this study revealed that WDFY4 is essential in anti-viral and anti-tumour immunity [30].

The same group published further research using CRISPR/Cas9 to inactivate *Wdfy4* in non-obese diabetic (NOD) mice [33]. cDC1s in NOD.*Wdfy4^−/−^* mice failed to cross-present cell-associated antigens to prime CD8+ T cells but remained capable of processing and presenting MHC-II restricted autoantigens, as well as activating pancreatic *β* cell-specific CD4+ T cells in lymph nodes, in agreement with their previous research.

Published in the same year, Liu and colleagues explored the function of WDFY4 in SLE [34]. Considering that *Wdfy4* expression was relatively higher in spleen and bone marrow in wild-type mice, as well as the critical role of B cells in SLE, a new *Wdfy4* B lymphocyte conditional knockout (*Wdfy4-*CKO) mouse model was generated. Compared with wild-type mice, *Wdfy4-*CKO mice showed decreased numbers of peripheral B cells, a defect in pro-B to pre-B cell transition in bone marrow and impaired immune response to antigen stimuli. A milder pristane-induced lupus phenotype was also observed in *Wdfy4-*CKO mice, including lower anti-dsDNA IgG, reduced immune complex deposition and inflammation in the kidney, and fewer spontaneous germinal centers in the spleen. By using small hairpin RNA-mediated stable knockdown of *WDFY4* in Raji cells, the authors also found that *WDFY4* deficiency could increase protein lipidation of microtubule-associated protein 1 light chain 3, suggesting that WDFY4 facilitates noncanonical autophagic activity in B cells [34].

Later, the same group reported research using the *Wdfy4^loxp/loxp^/Lck-*Cre^+/−^ CKO mouse model, focusing on T cells. Lack of *Wdfy4* in T cells was demonstrated to enhance reactive oxygen species-induced apoptosis via the p53 and ERK signalling pathways and suppress cell proliferation, resulting in a decrease in the number of peripheral CD8+ T cells and an impaired anti-tumour response [35]. WDFY4 deficiency was also confirmed to promote the differentiation of Th2 cells and the production of Th2 cytokines [36]. The authors established ovalbumin-induced asthmatic mouse models in both CKO and wild-type mice, demonstrating that *WDFY4* deficiency could promote Th2 cytokine production in the lungs and bronchoalveolar lavage fluid, and airway inflammation including inflammatory cell infiltration, goblet cell hyperplasia, mucus production, and collagen deposition, finally leading to airway remodeling.

A systematic genetic analysis study using a novel computational strategy, Gene-Module Association Determination, verified that *WDFY4* is associated with antigen processing, T cell activation, and immune response in human, mouse, and rat [37]. This analysis demonstrated that *WDFY4* is highly conserved across species and plays an essential role in the immune system.

## The role of *WDFY4* in idiopathic inflammatory myopathy

The idiopathic inflammatory myopathies (IIMs) are a group of heterogeneous autoimmune diseases, characterized by myositis, arthritis, specific skin rashes, interstitial lung disease (ILD), and other organ involvement. While the pathogenesis of IIMs hasn’t been fully elucidated and despite the heterogeneity among different myositis subgroups, previous studies suggest a similar pathogenic mechanism to other autoimmune diseases [38]. Environmental factors such as smoking, UV radiation, and viral infection may trigger excessive immune activation in genetically susceptible individuals. GWAS have reported genetic risk factors related to both HLA and non-HLA regions, in different myositis subgroups including myositis-specific autoantibody or myositis-associated autoantibody-positive subgroups [1, 39–41].

Clinically amyopathic dermatomyositis (CADM) patients usually show no or mild muscle involvement, typical skin rashes, and RP-ILD. The concept of CADM was initially proposed to distinguish the characteristics of this group of diseases from classic dermatomyositis [42]. Sato *et al.* detected a novel antibody targeting a 140kDa polypeptide in sera of CADM patients [43] and later identified the antigen as MDA5 [44] encoded by the RNA helicase *IFIH1* (Interferon-Induced Helicase C Domain-Containing Protein 1). However, the pathogenesis or genetics of CADM remained unclear.

In 2018, Kochi *et al.* first reported an intronic SNP rs7919656 in *WDFY4* in a Japanese CADM cohort [7]. GWAS was conducted in 576 Japanese IIM cases, including 33 CADM cases, and 6270 Japanese healthy controls. Although no associations were identified at genome-wide significance in the whole IIM cohort, polymyositis, or dermatomyositis groups, *WDFY4* rs7919656 was significantly associated with CADM (OR = 3.87, 95% CI 2.23–6.55, *p* = 1.5 × 10^−8^), albeit in a small sample size. The A allele frequency in CADM cases was 0.364, significantly higher than in controls (allele frequency = 0.129). Cis-eQTL analysis of *WDFY4* rs7919656 and variants in strong LD using the Guevadis project samples identified a splicing effect on the WDFY4 protein. An alternative exon extends exon 36, introduces an alternate stop codon and translates the original protein into a truncated isoform (tr-WDFY4) without BEACH or WD domains (Fig. 1B). Trans-eQTL analysis found that the expression of 279 genes was regulated by rs7919656, and weighted parametric gene set analysis identified the downstream transcription factors related to nuclear factor-kappa B (NF-κB) pathway. Further *in vitro* functional experiments demonstrated that both full-length WDFY4 and tr-WDFY4 interact with innate pattern recognition receptors including MDA5. Notably, tr-WDFY4 could markedly enhance the MDA5-induced apoptosis and alter type I interferon signalling (Fig. 2). In summary, this study not only proposed the mechanism of *WDFY4* function in immune cells, but also provided new clues for the pathogenesis of CADM as well as MDA5+DM [7].

Recently, Guo *et al.* [45] reported a genotyping study of SNP rs7919656 in Chinese IIM patients. Two hundred and fifty-four MDA5+DM cases, 53 anti-MDA5 negative dermatomyositis (MDA5-DM) cases, 72 anti-synthetase syndromes (ASyS) cases, and 192 healthy controls were included. No significant differences in allele frequency were observed in patients with MDA5+DM compared with healthy controls. However, genotypes with at least one minor allele (GA+AA) were significantly associated with RP-ILD in MDA5+DM patients (OR = 2.11, 95% CI 1.21–3.69, *p*= 0.007, total 71 cases), compared with the GG genotype (183 cases). Patients with minor allele A at rs7919656 also had higher one-year mortality (35.2%) than those with genotype GG (24.0%). In MDA5+DM patients, the expression of *WDFY4* in peripheral blood mononuclear cells (PBMC) was significantly higher in those with variant genotype GA+AA (*p*< 0.05). Furthermore, MDA5+DM patients with RP-ILD had higher *WDFY4* expression in PBMC than those without RP-ILD, as well as ASyS and MDA5-DM patients with ILD. Immunohistochemical staining revealed that WDFY4 protein was present extensively in the lung biopsy sample from a MDA5+DM-RP-ILD patient with GA genotype, compared with samples from another MDA5+DM patient without RP-ILD and a healthy control, both with wild-type GG. These clinical and laboratory data together suggested that the *WDFY4* variant could be a useful tool to predict the prognosis of Chinese MDA5+DM patients.

## Future research agenda for *WDFY4* in autoimmune diseases

Based on the large GWAS conducted across multiple autoimmune disease cohorts and recent findings from functional experiments, our understanding of the role of WDFY4 in the anti-viral and anti-tumour immune responses has markedly advanced in the past five years. Two recent studies on IIM (2, 10) provide new insights into the potential value of WDFY4 as a biomarker for disease prognosis. However, additional research is required to solve several issues in this field.

In the Kochi study, 21 European CADM cases recruited by the international Myositis Genetics Consortium and 84 matched controls were included for replication of the CADM-associated SNPs [7]. However, in European CADM cases, *WDFY4* rs7919656 did not show a significant signal, whilst two distinct (*r*^2^ < 0.2 with rs7919656) nearby SNPs were nominally associated with disease (rs11101462, *p* = 0.0092 and rs2889697, *p*= 0.0058). It would be beneficial to conduct GWAS studies in larger, multi-ancestry cohorts. The subsequent evidence in the clinical application is relatively scarce. Limited by the incidence of MDA5+DM and the number of accessible cases, the predictive value of the *WDFY4* polymorphism reported in the Chinese cohort did not achieve a significant difference in one-year survival analysis. It remains unclear whether the association between *WDFY4* and RP-ILD in MDA5+DM patients is unique to the East Asian population or whether different *WDFY4* variants have a similar role in other populations. Meanwhile, there is insufficient evidence to establish an association between WDFY4 and specific clinical phenotypes of other autoimmune diseases, such as SLE or RA-related ILD. Through international collaboration, larger cohorts with clinical response outcome data are required to further evaluate these findings. Also, the functions of WDFY4 and its role in the pathogenesis of autoimmune diseases haven’t been fully illustrated. Further research is needed to elucidate how the variants discovered through GWAS affect protein function or cause disease.

## Conclusion

GWAS identified and confirmed the association of multiple *WDFY4* variants in different ancestry populations with autoimmune diseases, especially SLE, RA, and PBC. Functional studies in recent years have confirmed that WDFY4 plays a role in anti-infection and anti-tumour immunity through cross-presentation. A recent study in the Japanese population found a new variant rs7919656 that can lead to truncated WDFY4 protein and is related to CADM. In vitro experiments confirmed that the truncated isoform can also interact with MDA5 and induce apoptosis. Later rs7919656 was reported to be associated with RP-ILD in Chinese MDA5+DM patients. Considering the high correlation between RP-ILD and poor disease outcome, as well as the limitation in early diagnosis of RP-ILD, this variant could be a promising predictor of mortality in RP-ILD. Further studies are required to validate the role of WDFY4 in autoimmune diseases.

## Supplementary Material

keae183_Supplementary_Data

## Data Availability

The data underlying this article are available in the article.
