# Supplementary material for: The clinical relevance of WDFY4 in autoimmune diseases in diverse ancestral populations
Source: Rheumatology (Oxford). 2024 Mar 20;63(12):3255–62. doi: 10.1093/rheumatology/keae183 (PMC11637422; doi:10.1093/rheumatology/keae183)
Supplement: keae183_Supplementary_Data [file keae183_supplementary_data.zip › keae183_Supplementary_Data/rhe-23-1975-File005.docx]

Supplementary Table S1: main splice variants of WDFY4*, based on Ensembl(1) and RefSeq(2).

| accession in Ensembl | | accession in RefSeq | | Transcript length(bp) | protein size(aa) |
| --- | --- | --- | --- | --- | --- |
| transcript ID | translation ID | transcript ID | translation ID |  |  |
| ENST00000325239.12** | ENSP00000320563.5 | NM_001394531.1 | NP_001381460.1 | 10082 | 3184 |
| ENST00000360890.6 | ENSP00000354141.2 | NM_001370154.1 | NP_001357083.1 | 2193 | 654 |
| ENST00000490507.1 | ENSP00000491967.1 | - | - | 428 | 77 |
| - | - | NM_001370153.1 | NP_001357082.1 | 5637 | 1614 |
| - | - | NM_020945.2 | NP_065996.1 | 10023 | 3184 |

*Only protein coding transcripts and no predicted transcripts are listed. ‘-’ means the same transcript were not matched in the other database. ** This is the MANE select (3)transcript.

References:

1 Martin FJ, Amode MR, Aneja A et al. Ensembl 2023. Nucleic Acids Res 2023;51:D933–41.

2 Maglott D, Ostell J, Pruitt KD, Tatusova T. Entrez gene: gene-centered information at NCBI. Nucleic Acids Res 2011;39:D52–7.

3 Morales J, Pujar S, Loveland JE et al. A joint NCBI and EMBL-EBI transcript set for clinical genomics and research. Nature 2022;604:310–5.
