# Supplementary material for: The clinical relevance of WDFY4 in autoimmune diseases in diverse ancestral populations
Source: Rheumatology (Oxford). 2024 Mar 20;63(12):3255–62. doi: 10.1093/rheumatology/keae183 (PMC11637422; doi:10.1093/rheumatology/keae183)
Supplement: keae183_Supplementary_Data [file keae183_supplementary_data.zip › keae183_Supplementary_Data/rhe-23-1975-File006.docx]

Supplementary Figure S2:


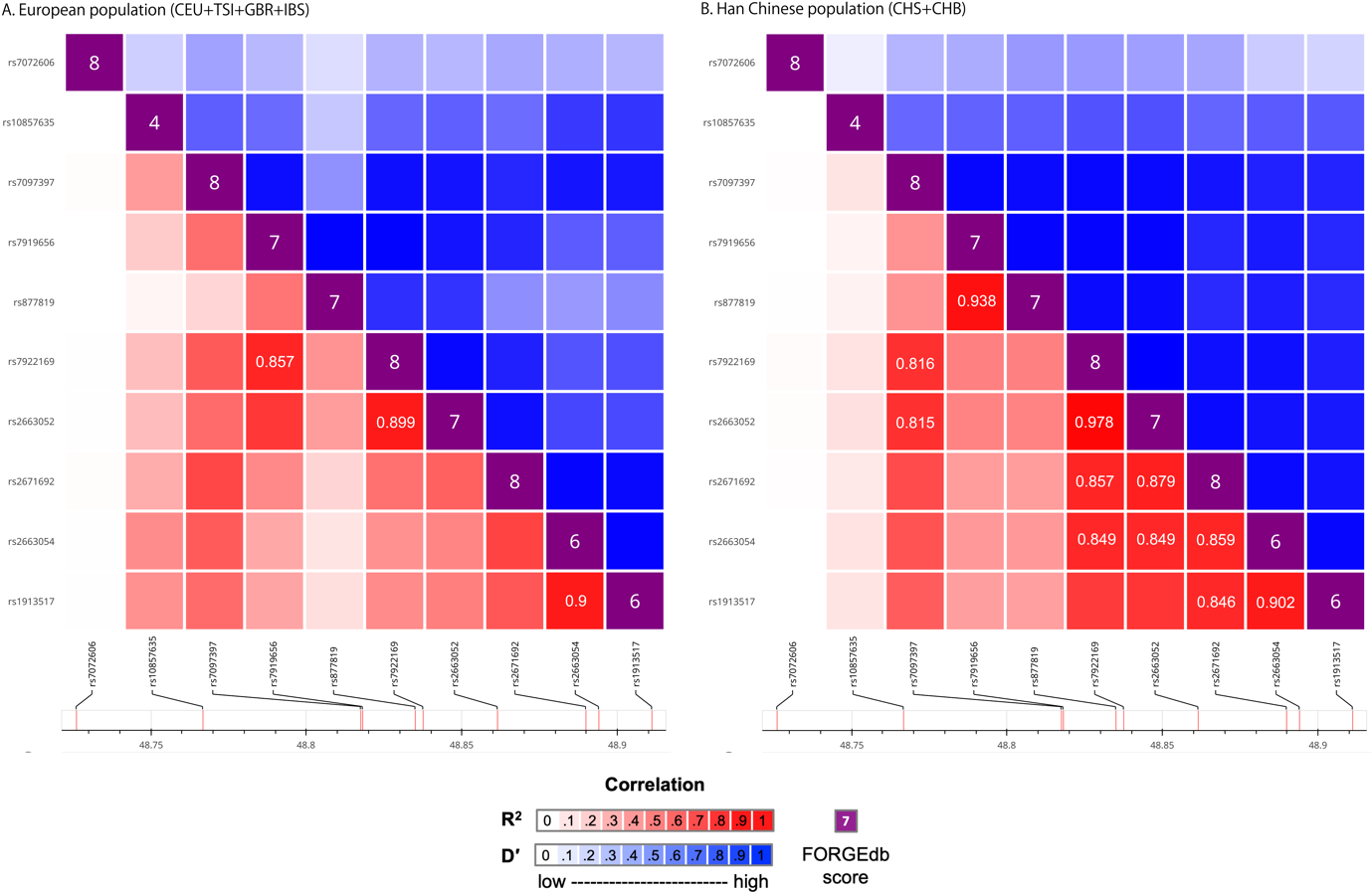


Supplementary Figure S2: Linkage disequilibrium (LD) among 10 WDFY4 SNPs related to autoimmune diseases, generated based on LDmatrix module(1) and GRCh38 1000 Genome Project dataset (2). A. LD plot for 4 European sub-populations, including Utah Residents from North and West Europe (CEU), Toscani in Italia (TSI), British in England and Scotland (GBR) and Iberian population in Spain (IBS). B. LD plot for Han Chinese in Beijing (CHB) and Southern Han Chinese (CHS). R^2^: a measure of correlation of alleles for two variants. D’: an indicator of allelic segregation for two variants. FORGEdb score(3): predictor for the regulatory potential of genetic variants, ranging from 0 to 10. R^2^ values greater than 0.8 are marked on the corresponding squares in the heatmap.

References:

1 Machiela MJ, Chanock SJ. LDlink: a web-based application for exploring population-specific haplotype structure and linking correlated alleles of possible functional variants. Bioinformatics 2015;31:3555–7.

2 Odhams CA, Cortini A, Chen L et al. Mapping eQTLs with RNA-seq reveals novel susceptibility genes, non-coding RNAs and alternative-splicing events in systemic lupus erythematosus. Hum Mol Genet 2017;26:1003–17.

3 Breeze CE, Haugen E, Gutierrez-Arcelus M et al. FORGEdb: a tool for identifying candidate functional variants and uncovering target genes and mechanisms for complex diseases. Genome Biol 2024;25:3.
